# Using population viability analysis, genomics, and habitat suitability to forecast future population patterns of Little Owl *Athene noctua* across Europe

**DOI:** 10.1002/ece3.3629

**Published:** 2017-11-12

**Authors:** Line Holm Andersen, Peter Sunde, Irene Pellegrino, Volker Loeschcke, Cino Pertoldi

**Affiliations:** ^1^ Department of Bioscience Aarhus University Aarhus Denmark; ^2^ Department of Science and Technological Innovation University of Piemonte Orientale Alessandria Italy; ^3^ Section of Biology and Environmental Science, Department of Chemistry and Bioscience Aalborg University Aalborg Øst Denmark; ^4^ Aalborg Zoo Aalborg Denmark

**Keywords:** conservation, habitat suitability, management, minimum viable population size, population viability analysis, RAMAS/GIS, VORTEX

## Abstract

The agricultural scene has changed over the past decades, resulting in a declining population trend in many species. It is therefore important to determine the factors that the individual species depend on in order to understand their decline. The landscape changes have also resulted in habitat fragmentation, turning once continuous populations into metapopulations. It is thus increasingly important to estimate both the number of individuals it takes to create a genetically viable population and the population trend. Here, population viability analysis and habitat suitability modeling were used to estimate population viability and future prospects across Europe of the Little Owl *Athene noctua*, a widespread species associated with agricultural landscapes. The results show a high risk of population declines over the coming 100 years, especially toward the north of Europe, whereas populations toward the southeastern part of Europe have a greater probability of persistence. In order to be considered genetically viable, individual populations must count 1,000–30,000 individuals. As Little Owl populations of several countries count <30,000, and many isolated populations in northern Europe count <1,000 individuals, management actions resulting in exchange of individuals between populations or even countries are probably necessary to prevent losing <1% genetic diversity over a 100‐year period. At a continental scale, a habitat suitability analysis suggested Little Owl to be affected positively by increasing temperatures and urban areas, whereas an increased tree cover, an increasing annual rainfall, grassland, and sparsely vegetated areas affect the presence of the owl negatively. However, the low predictive power of the habitat suitability model suggests that habitat suitability might be better explained at a smaller scale.

## 1 INTRODUCTION

Human land use alters natural landscapes all over the world, and in Europe, 75% of all land is covered by either rural or urban areas, the main land uses being agriculture and forestry (FAO, 2014). Farmland areas traditionally support a large number of species living in or in close proximity to farmland areas (Fuller, 2000). But over the last 60 years, the agricultural landscape has undergone a substantial change in order to increase productivity (Fuller, 2000). The habitat changes have led to a severe decline in several species associated with the agricultural landscape, and among farmland birds, the main drivers of the decline have been habitat fragmentation, agricultural intensification, and land abandonment (Fuller et al., 1995; Newton, 2004; Sotherton, 1998).

Habitat fragmentation might divide populations that were once continuous into either metapopulations, populations that occasionally exchange individuals and genes, or to completely disconnected populations with no gene exchange at all. A metapopulation consists of several populations connected by dispersal. Local populations might go extinct, but the patches remain part of the metapopulation due to the chance of recolonization (Hanski & Gilpin, 1991). When making management decisions it is important to determine whether the individual subpopulations are demographically and/or genetically viable on their own or as part of a metapopulation. In order to forecast the outcomes of different management scenarios including the status quo, estimates of minimum viable population size (MVP) and minimum carrying capacity that can sustain the population demographically and genetically are essential decision tools. Population viability analysis (PVA) can be used to estimate the viability, the MVP, and extinction risk of populations (Boyce, 1992). PVAs are becoming increasingly sophisticated, and different softwares can encompass many types of information alongside life history data, including genetic data (VORTEX), spatial information (RAMAS), species interactions, and disease spread (Akcakaya, 2000; Andersen, Sunde, Loeschcke, & Pertoldi, 2015; Bradshaw et al., 2012; Larue & Nielsen, 2016; Olsen et al., 2014; Prowse et al., 2013).

One species affected by the changed agricultural regime is the Little Owl *Athene noctua* (Framis, Holroyd, & Manosa, 2011; Thorup, Sunde, Jacobsen, & Rahbek, 2010). The Little Owl has a wide range extending from northern Africa to southern Scandinavia and from the west coast of Europe to the east coast of Asia (Cramp, 1977; Génot, Juillard, & Nieuwenhuyse, 1997). Several subspecies of the Little Owl exist, and population genetic studies suggest that several genetic populations can be found in Europe (Cramp, 1977; Pellegrino, Boatti et al., 2015; Pellegrino, Negri et al., 2015). It can be found in many types of habitats including agricultural fields, orchards, open woodland, and steppes and tend to avoid closed forest and heavily buildup areas (Exo, 1992; Génot et al., 1997; Gottschalk, Ekschmitt, & Wolters, 2011; Nieuwenhuyse & Bekaert, 2002; Tome, Catry, Bloise, & Korpimaki, 2008; Zabala et al., 2006). The Little Owl is sedentary, and juveniles usually settle within 20 km of their nesting place (Bønløkke et al., 2006; Nieuwenhuyse, Génot, & Johnson, 2008). Little owls depend on cavities for breeding, and lack of breeding cavities is known to limit population growth (Exo, 1992; Nieuwenhuyse et al., 2008). Another factor presently limiting the species is the decreasing area of suitable habitat for both feeding and breeding (Šálek & Schröpfer, 2008; Santos & Suarez, 2005; Thorup et al., 2010; Zmihorski, Altenburg‐Bacia, Romanowski, Kowalski, & Osojca, 2006; Zmihorski, Romanowski, & Osojca, 2009).

Using the Little Owl as a model species, we aim at providing information valuable for the management of Little Owl populations across Europe. We achieve this aim by compiling information on both population viability, genetic viability, and habitat suitability. First, population and genetic viability are estimated using the PVA program VORTEX. We aim to estimate whether the populations corresponding to the genetic units defined by Pellegrino, Boatti et al. (2015) are sufficiently large to ensure long‐term population viability. In order to do this, we will estimate the genetic MVP of each of the six population clusters and compare the MVP to the actual population sizes. If the MVP is equal to or smaller than the actual population size, the population may be considered genetically viable over the next 100 years. A time frame of 100 years is commonly used for PVA and is suitable for organisms with shorter lifespans (Boyce, 1992; Murn & Botha, 2017; Walters, Crowder, & Priddy, 2002). If the MVP is larger than the actual population size, the population may not be viable. Second, we wish to look at Little Owl distribution at a macroscopic landscape scale across the European range. Using habitat suitability analysis, we aim to investigate the factors most important for the presence of the Little Owl at a European scale. Finally, we used the PVA program RAMAS is used to predict the possible future distribution and population trend of the Little Owl within Europe when including demographic knowledge alongside spatial information.

## 2 MATERIALS AND METHODS

The materials and methods section is subdivided into several sections. The first section deals with the preparation of the already existing genomic dataset. The second section deals with the PVA conducted in VORTEX, in which the genomic dataset is used. Third, the habitat suitability analysis is described. Finally, the fourth section describes the PVA conducted in RAMAS, in which spatial data are included.

### 2.1 Genomic data preparation

The genomic dataset described in Pellegrino, Boatti et al. (2015) includes genomic data from 53 individual Little Owls from Europe. The dataset was reduced, creating a dataset including only loci under directional selection (6,894 SNPs). We used the program GenePop 4.4.3 (Rousset, 2008) to find the allelic frequencies within each population defined in the study of Pellegrino, Boatti et al. (2015). These populations were a Portuguese, a Spanish, a French–Danish–the Netherlands population cluster (in this region Little Owls occur in a number of more or less isolated populations, but for convenience, it will be termed “population” in the following), an Italian population divided into a southern and northern population and a Greek–Romanian–Cypriote population.

### 2.2 VORTEX simulations

VORTEX is a PVA software that enables the user to include genomic datasets and model‐predicted changes in the genomic layout along with population trends. A PVA was conducted in VORTEX 10.0.7.0 (Lacy, Miller, & Traylor‐Holzer, 2014; Lacy & Pollak, 2013).

Each simulation was repeated 10 times and ran over a time span of 100 years.

#### 2.2.1 Populations and study area

In both the VORTEX and RAMAS (see later) simulations, the European populations of Little Owl were modeled as a metapopulation, consisting of populations and subpopulations that to a varying degree are connected as a function of distance.

The populations simulated in the VORTEX analysis consisted of several large populations defined by their genetic structure (Section 2.2.2). The western European population is estimated to contain 198,000–638,000 individuals (Spain 80,000 individuals, Portugal 116,000–274,000 individuals, France–Denmark–the Netherlands 56,000–118,000 individuals). The total Italian population counts 80,000–140,000 individuals. The Balkan population is estimated to count 48,000–130,000 individuals (Birdlife International, 2015). For each of the simulated populations, life history parameters were estimated individually (Table 1).

**Table 1:** Parameters used in the simulations in VORTEX

Parameter	Value (IT, BK, ES, PT, and NE)	Reference
Number of iterations	10	
Adult mortality (aged 1‐death)^a^	35%, 35%, 35%, 35%, and 36.7%	Nieuwenhuyse et al. (2008), Thorup et al. (2010)
Juvenile mortality (aged 0–1)^a^	70%, 70%, 70%, 70%, and 80%	
Environmental correlation in mortality rates	5 (adult), 10 (juvenile)	
Mating structure	Short‐term monogamous	Nieuwenhuyse et al. (2008)
Breeding age	1	Juillard (1984), Nieuwenhuyse et al. (2008)
Maximum age of reproduction	15	Nieuwenhuyse et al. (2008)
Density dependency	Yes	
Mean number of progeny per brood	4.64, 5.24, 4.4, 3.3, and 3.78	Table S5
SD, mean number of progeny	1.0, 1.0, 1.0, 1.2, and 1.0	
Maximum number of progeny	10, 7, 10, 5, and 10	Nieuwenhuyse et al. (2008)
Ratio of breeding pairs successful in getting fledglings	85%	Tome, Bloise, and Korpimaki (2004), Jacobsen (2006)
Number of breeding attempts per year	1	Nieuwenhuyse et al. (2008)
Sex ratio at birth (males:females)	50:50	
Number of males in breeding pool	100%	
Number of females in breeding pool	100%	
Minimum age of dispersal^b^	1	Cramp (1977), Pedersen, Thorup, Sunde, Jacobsen, and Rahbek (2013)
Maximum age of dispersal^b^	3	
Probability of dispersal^b^	5%	
Dispersing sex^b^	Both (70% survive dispersal)	
Population size	Variable in order to find MVP	
Catastrophe 1: Cold winter	Rate of 5%, survival 75% of normal	Poulsen (1940, 1957), Dobinson and Richards (1964)
Catastrophe 2: High rainfall	Rate of 5%, reproduction 75% of normal	Bultot et al. (2001), Nieuwenhuyse et al. (2008)

The numbers are given for the Italian population first (IT), followed by the Balkan population (BK), the Spanish population (ES), the Portuguese population (PT), and last the northern European population (NE). If the same value is used for all populations, only one value is listed.

a For the populations IT, BK, ES, and PT, both a high and a low mortality rates were simulated. The lower adult mortality rate is 35%, and the high is 38%, whereas the low juvenile mortality rate is 70%, and the high is 75%.

b Dispersal only applicable when more than one population is simulated.

The MVP is here defined as the minimum population size that maintains at least 95%–99% of the initial genetic diversity over a 100‐year period. In order to find the minimum initial population size that retained 95%–95% genetic diversity, the populations were modeled with a range of different initial population sizes at a constant carrying capacity K. The initial population size was increased/decreased until the minimum initial population size was found. For populations with a positive stochastic growth rate, K was altered in order to find the minimum viable K (MVP(K)) for maintaining 99% and 95% genetic diversity, respectively.

#### 2.2.2 Carrying capacity

A theoretical K was estimated for all regions included in the simulations. It was estimated from data on population density and the area of interest (Table S1). This K was used in the simulation where the initial population size was varied.

#### 2.2.3 Density dependency

Populations of Little Owl breed in a density‐dependent manner, as isolated pairs lay larger clusters (Bultot, Marié, & Van Nieuwenhuyse, 2001). In order to include this in the simulations, a density dependence function was built on the suggested function for density dependence listed by VORTEX (Lacy et al., 2014):

(1) $$\text{MO}\left(N\right)\;=\;\text{MO}\left(0\right)\;-\;\left(\text{MO}\left(0\right)\;-\;\text{MO}\left(K\right)\right)\cdot {\left(N/K\right)}^{2}\cdot \left(\frac{N}{N-1}\right)$$

MO(*N*) is the mean number of offspring at population size *N*. MO(0) is the mean number of offspring produced at low densities. *K* is carrying capacity.

#### 2.2.4 Catastrophes

In VORTEX, environmental stochasticity can be modeled by adding catastrophic events to the model. A catastrophe occurs at a certain risk every year and might affect reproduction, survival, or both. Cold winters have a negative impact on the survival of Little Owls (Dobinson & Richards, 1964; Nieuwenhuyse et al., 2008; Poulsen, 1940, 1957). In the simulations, a cold winter had a 5% risk of occurring every year in the northern European population. When occurring, the survival rate was lowered to 75% of the normal rate. High rainfall also affects the Little Owl and decreases the reproductive success (Bultot et al., 2001; Nieuwenhuyse et al., 2008; Tome et al., 2008). High rainfall occurred at a 5% risk every year in all populations and reduced the number of offspring produced to 75%.

#### 2.2.5 Mortality rates

The mortality rate differs greatly among juvenile birds (<1‐year old) and adult birds, and between populations. Therefore, population‐specific mortality rates were used if available (Table S2). When population‐specific mortality rates could not be obtained, simulations were run both at a lower mortality rate (Juvenile mortality = 70%, Adult mortality = 35%) and at a higher mortality rate (Juvenile mortality = 75%, Adult mortality = 38%).

#### 2.2.6 Genetics

Genomic data were included in the VORTEX simulations (Pellegrino, Boatti et al. 2015). The genomic dataset was used to estimate the genomic development of the population under the simulated conditions. VORTEX made it possible to estimate whether genomic data were likely to be lost or not over the course of 100 years.

### 2.3 Habitat suitability analysis

#### 2.3.1 Study area

The habitat suitability was studied within a subset of Europe, including the following: Albania, Austria, Belgium, Bosnia and Herzegovina, Croatia, Czech Republic, Denmark, Estonia, France, Germany, Hungary, Italy, Ireland, Kosovo, Latvia, Lithuania, Macedonia, Moldova, Montenegro, the Netherlands, Poland, Portugal, Romania, Serbia, Slovakia, Slovenia, Spain, Switzerland, Turkey, and the United Kingdom.

#### 2.3.2 Regression analysis

A general linear model (GLM) was performed in R in order to determine the explanatory variables that had a significant influence on the presence–absence of the Little Owl. General linear models (GLM) can be used to determine the environmental factors that shape a species’ distribution and thereby quantify the species niche (Austin, Nicholls, & Margules, 1990; Vetaas, 2002). The datasets used in the GLM are described below and include both climatic data, data on habitat characteristics, and information on human activities.

In order to determine the factors most influential on the distributional pattern of the Little Owl on a macroscopic landscape scale, the probability of presence in 5 × 5 km squares was modeled with logistic regression analysis as a function of the data described below (Franklin, 1995). All continuous variables were checked for normality. If a variable was not normally distributed, it was log transformed.

To avoid including highly correlated variables in the regression, both the Pearson correlation and the Variance Inflation Factor (VIF) were calculated using R 3.1.2. The VIF reports how much of the variability of a given explanatory value is already explained in the model due to correlation (Craney & Surles, 2002).

#### 2.3.3 Data

**Figure 1:**
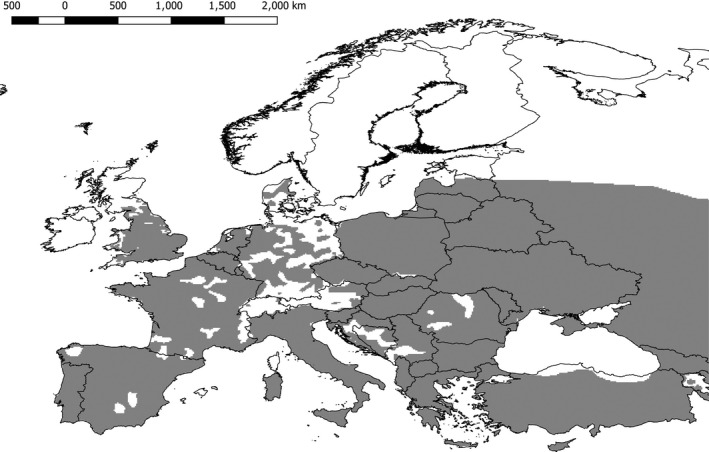
European distribution map of the Little Owl *Athene noctua* used on the RAMAS simulations. Gray is present, white is absent. Within Europe, the Little Owl is native to all but Great Britain, where it has been introduced

All the following datasets were rescaled to a 5‐km resolution using ArcGIS 10.3.1 (ESRI, 2015). A presence–absence dataset of the worldwide distribution of the Little Owl was provided by the IUCN (2 × 2 km original resolution; BirdLife International & NatureServe, 2014; Figure 1). A dataset containing long‐term average climatic data for a number of climatic factors was included (original resolution 0.93 × 0.93 km at the Equator, average values for the years 1950–2000; Hijmans, Cameron, Parra, Jones, & Jarvis, 2005). The climatic parameters included were annual mean temperature (BIO1, ͦ C*10), temperature seasonality (BIO4, defined as the standard deviation*100), minimum temperature of the coldest quarter (BIO11, C*10), annual precipitation (BIO12, mm, log transformed), and precipitation of the wettest month (BIO13, mm, log transformed). Altitude was also included (ALT, m above sea level). Different habitat types were included as follows: forest (FOR), arable land (ARA), urban and industrial areas (URB), permanent crops and pastures (including orchards; PCR), mixed agricultural areas (MAG), water bodies (WAT), sparse vegetation (SVE), and grassland (GRA; CORINE data, 250 × 250 m original resolution; European Environment Agency, 2013; Table S3 and Fig. S1). The Human Footprint, featuring information on human population pressure, human land use and infrastructure, and human access (including roads), was also taken into account (original resolution 1 × 1 km; Sanderson et al., 2002; Wildlife Conservation Society—WCS and Center for International Earth Science Information Network—CIESIN—Columbia University, 2002).

#### 2.3.4 Habitat suitability function

Habitat suitability (HS) modeling uses information on a species dependency on the habitat to predict the likelihood of occurrence in a given area based on environmental variables (Franklin, 1995; Hirzel & Le Lay, 2008; Pereira & Itami, 1991). It describes the quantitative relationship between the physical and biological factors in a given environment, and further the suitability of the given habitat for a specific species (Akçakaya & Root, 2013). We projected the model to the entire area of interest using Equation 2. As a logistic regression (*y*, Equation 3) was used to describe presence/absence of the Little Owl, the logit link function (Equation 2) describes the probability (*p*) that the owl is present (Akçakaya & Root, 2013):

(2) $$p\;=\;\frac{1}{\exp\left(-y\right)+1},\;\;\text{where}$$

(3) $$y\;=\;\beta \;+\;\beta_{1}\cdot x_{1}\;+\;\beta_{2}\cdot \;x_{2}\;+\cdots +\;\beta_{n}\cdot \;x_{n}$$

### 2.4 RAMAS/GIS and RAMAS Metapop

Using the population viability software RAMAS/GIS and RAMAS Metapop (Akçakaya & Root, 2013), a PVA was conducted on the Little Owl on a continent scale with the aim of evaluating the population trend and the future distribution of the species in different European regions and on the continent as a whole. The RAMAS software makes it possible to include spatial data into the PVA. RAMAS/GIS was used for modeling the initial species distribution, and RAMAS Metapop incorporated both the initial distribution found in RAMAS/GIS and included population‐specific life history data and stochastic events.

Each simulation was repeated 100 times over a time span of 100 years. As described below, the simulated scenarios tested population viability under varying initial population sizes and survival rates.

#### 2.4.1 Study area

The same study area was used in the RAMAS/GIS analysis as in the Habitat suitability analysis.

#### 2.4.2 Initial distribution, population size, and K

The initial distribution map was provided by the BirdLife International and the IUCN Red list (Figure 1; BirdLife International & NatureServe, 2014). RAMAS/GIS was used to estimate the number and locations of populations within the European population (Table S4 and Fig. S3). Using the geographical location of each population, the population was assigned a low and a high initial population size on basis of the population count data from BirdLife International (2015; Table S4). An estimated 1,060,698 to 2,233,635 Little Owls can be found within the study area (BirdLife International, 2015). As RAMAS/Metapop assumes exponential population growth until K is met, K was set to be equal to the initial population size.

#### 2.4.3 Stage matrix

A stage‐structured model of survival and fecundity was used. The stages included were a fledgling (0‐ to 1‐year olds), a juvenile (1‐ to 2‐year olds), and an adult stage (2 and older). A total of 83% of all juveniles were assumed to reproduce, while 100% of the adults were assumed to reproduce (Møller, 2006; Nieuwenhuyse et al., 2008).

When calculating the mean fecundity on basis of nest surveys, it is important to take the survival rates into account (Akçakaya, 2000). A Little Owl lays on average 3.87 eggs (Table S5), and on average, 40.14% of these will fail in surviving to fledgling stage (Nieuwenhuyse et al., 2008). This results in a mean fecundity of 2.32 fledglings. The mean fecundity was used in all populations for which the actual fecundity had not been recorded. When possible, the actual fecundity was used (Table S2, observed clutch size times 0.598). The same applies for the survival rates (Table S2). If no specific survival rate was known, the mean survival rate was used (Fledglings 26.8%, juvenile and adult owls 67.0%; Table S2).

When the actual survival rate was unknown, simulations were run with the mean survival rates, with a lowered survival (25% for fledglings, 62% for juveniles and adults) and an increased survival (30% for fledglings, 70% for juveniles and adults).

#### 2.4.4 Dispersal

Dispersal in RAMAS/GIS is defined as follows:

(4) $$M_{ij}\;=\;a\exp\left(-D_{ij}^{c/b}\right)\;\text{if}\;D_{ij}\leq \;D_{\max}$$

(5) $$0\;\text{if}\;D_{ij}>D_{\max}$$

*a*,* b*, and *c* are input parameters, and *D*_max_ is the maximum recorded dispersal distance (Akçakaya & Root, 2013). In RAMAS/GIS, a distance matrix was produced (using edge‐to‐edge distances). The distance matrix and the formula above were used to calculate dispersal distances. In Little Owls, ringing data have recorded the maximum dispersal distance of juvenile owls to 182, 190, 220, 230, 270, and 600 km (Nieuwenhuyse et al., 2008). *D*_max_ is thus set at 250 km. The average dispersal distance of the Little Owl was calculated to 8.7 km (based on the average dispersal distance in Table 10.5 in Nieuwenhuyse and Bekaert (2002) and Bønløkke et al. (2006)). The factor *a* was set to 0.15, *b* to 6, and *c* to 1 (Fig. S2). Dispersal is assumed to happen 20 times more often in juvenile birds compared to adults. Dispersal was assumed to be independent of population density.

#### 2.4.5 Density dependency

The Ceiling density type was chosen (exponential growth until carrying capacity (the ceiling) is reached), and Allee effects included. Allee effects describe the situation where individual fitness is correlated with population density (Courchamp, Clutton‐Brock, & Grenfell, 1999; Stephens, Sutherland, & Freckleton, 1999). The Allee parameter was set to 100 if *K* > 100, meaning that at population densities of 100 individuals or less, the fecundity and survival will be halved. The Allee parameter was reduced to 10 if *K* < 100. Density dependency was set to affect all life stages and to affect both fecundity and survival.

#### 2.4.6 Catastrophes

Environmental stochasticity can be modeled by adding catastrophic events to the model. A catastrophe occurs at a certain risk every year and affects the vital rates of Reproduction and Survival to the same extent as described under catastrophes in VORTEX. Catastrophic events could happen in consecutive years, and their occurrences were correlated between populations.

## 3 RESULTS

### 3.1 VORTEX simulations

Positive stochastic growth rates were found in the Balkan population, the Italian populations, and the Spanish population (Table 2). In the northern European population and the Portuguese population, the stochastic growth rate was negative (Table 2).

**Table 2:** The mean stochastic growth rate for each of the simulated growth rates when keeping K constant and varying the initial population size

Population	Mortality rates	*r* (mean)
Portugal	Low	−.0102
Portugal	High	−.1095
Spain	Low	.0941
Spain	High	.0362
Northern Europe	Population‐specific	−.0793
Central Italy	Low	.1173
Central Italy	High	.0444
Northern Italy	Low	.1169
Northern Italy	High	.0452
Balkan	Low	.1988
Balkan	High	.2202

It was not possible to estimate the MVP(K) for the northern European population due to the negative growth rate of the population. The initial population would have to count more than 250,000 individuals to secure a loss of <5% genetic diversity, while more than 500,000 individuals are required to maintain 99% of the genetic diversity. As with the northern European population, the population growth rate of the Portuguese population was negative and an MVP(K) could not be estimated. The Portuguese population was very sensitive to changes in the mortality rates, and an increase in mortality from 70% to 75% in juveniles and from 35% to 38% in adults greatly increased the risk of extinction within a 100‐year period. The MVP was only estimated for populations with a positive or stable growth rate. The average MVP (99%) with regard to the carrying capacity for these populations was 4,700 individuals (low mortality rate) or 16,625 individuals (high mortality rate; Table 3). In order to sustain 95% genetic diversity, a K of 1,000 individuals was needed when simulating lower mortality rates, while on average 1,375 individuals were needed at high mortality rates (Table 3). The Balkan population did not seem particularly sensitive toward an increased mortality rate. When altering the K, it was found that a maximum of 6,500 individuals was necessary to maintain 99% of the total genetic diversity, while <1,000 individuals were needed to preserve 95% genetic diversity over 100 years. Both the Central and northern Italian populations have stable growth rates. Depending on the mortality rates of the populations, a K ranging from 5,000 to 30,000 individuals is needed to maintain 99% genetic diversity in the northern Italian population, whereas 4,500–10,000 individuals were sufficient for the Central Italian population. The Spanish population requires <1,000 individuals to maintain 95% genetic diversity, while estimated 4,500–20,000 individuals should be needed to maintain 99% genetic diversity depending on the mortality rates.

**Table 3:** The minimum *K* supporting a MVP that retains 95%/99% genetic diversity over 100 years

Population	Low mortality		High mortality	
	*K* (MVP_95%_)	*K* (MVP_99%_)	*K* (MVP_95%_)	*K* (MVP_99%_)
Balkan	1,000	4,500	1,000	6,500
Italy N	1,000	5,000	1,000	30,000
Italy C	1,000	4,500	1,000	10,000
Spain	1,000	4,500	1,000	20,000
Italy (C and N)	2 × 500	2 × 2,500	2 × 1,000	2 × 4,000
Mean	1,000	4,700	1,000	16,625

The minimum *K* was found for populations with a positive stochastic growth rate. In all cases, the initial population size was 1,000 individuals.

Simulating a metapopulation instead of a continuous population only increased the risk of extinction.

### 3.2 Habitat suitability analysis

**Figure 2:**
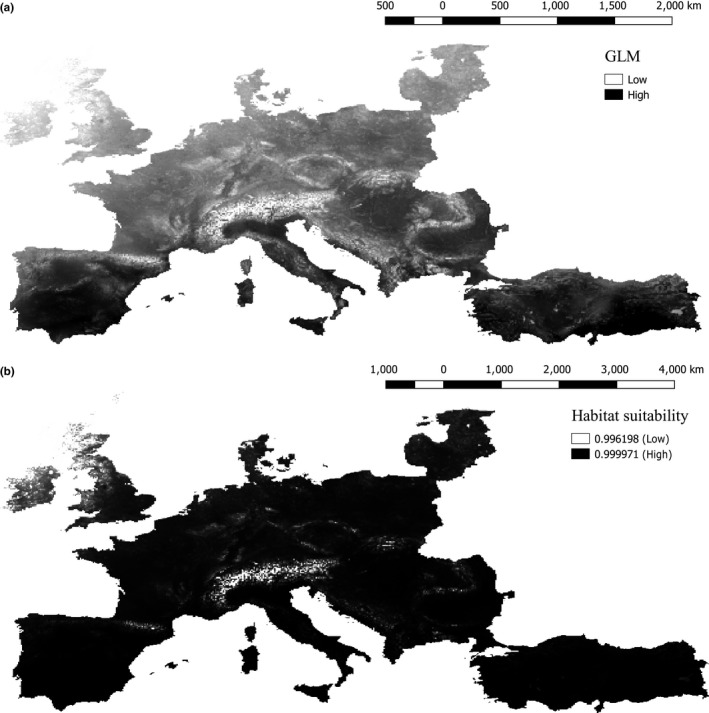
The habitat suitability for the Little Owl in Europe. (a) shows the results of the GLM, depicting the areas with low and high probability of finding the Little Owl. (b) shows the Habitat suitability map, with values ranging from 0 to 1, 1 being very suitable habitats, 0 being unsuitable habitats

The mean temperature of the coldest month (BIO11) was highly correlated with several of the other explanatory variable and was therefore eliminated from further analysis. After eliminating BIO11, all variables had VIF values below 10 and 15 explanatory variables remained. A model with all 15 explanatory variables was chosen for further analysis in R, where 10 were found significant (Table 4, Figure 2a). The species was limited by the proximity to inland water bodies, sparsely vegetated areas, grassland area, increasing tree cover, and increasing annual precipitation. Whereas the annual mean precipitation had a negative influence on the probability of presence, precipitation of the wettest month had a positive influence on the probability of presence of Little Owls. The annual mean temperature and temperature seasonality also had a positive influence on the presence of the Little Owl, along with the presence of urban/industrial areas and altitude.

**Table 4:** Coefficient estimates and standard errors, of the final little owl distribution model in Europe

	Coefficient	Standard error	*p*
Intercept	**6.5710000**	**0.360000**	**<2e**−**16** ***
Permanent crops	0.0439200	0.029140	.1317
Water	−**0.3748000**	**0.054000**	**3.91e**−**12** ***
Forest	−0.0360000	0.024530	.1422
Sparse vegetation	−**0.1225000**	**0.061150**	**.045194** *
Arable land	0.0715700	0.026070	.006054
Grassland	−**0.1138000**	**0.033360**	**.000647** ***
Mean human footprint	0.0012460	0.078130	.1106
Precipitation, wettest month (log)	**0.4989000**	**0.072980**	**8.11e**−**12** ***
Annual precipitation (log)	−**2.0970000**	**0.081740**	**<2e**−**16** ***
Mean tree percentage	−**0.0164900**	**0.000455**	**<2e**−**16** ***
Temperature seasonality	**0.0004870**	**0.000009**	**<2e**−**16** ***
Altitude	**0.0010080**	**0.000022**	**<2e**−**16** ***
Annual mean temperature	**0.0393200**	**0.004138**	**<2e**−**16** ***
Urban/industrial area	0.2090000	0.045070	3.51e−06***
Mixed agriculture	−0.0250200	0.038200	.5125

Significant variables in the model are highlighted in boldface. The significance codes for the *t* value are as follows: ***0.001; *0.05.

As illustrated by the habitat suitability map (Figure 2b), the HS function predicts a large area of potentially suitable habitat for the Little Owl, excluding high mountainous areas (The Alps and The Pyrenees), northern Great Britain, the north of Denmark, and in Latvia.

### 3.3 RAMAS/GIS and RAMAS Metapop

Regardless of the initial population size and mortality rate, the abundance of the metapopulation decreased over the course of 100 years. Patch occupancy dropped continuously when simulating low survival and stabilized after 25 years when simulating high survival (Fig. S4). The initial population size did affect the number of patches occupied after 100 years (Fig. S4).

**Figure 3:**
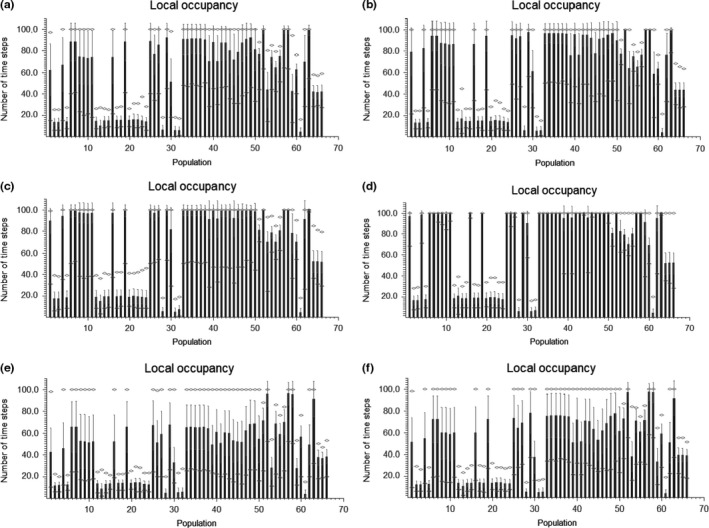
The local population occupation duration. The figure shows the number of time steps a given population was occupied during the 100‐year period. The mean of all replications, along with the std. average, and the minimum and maximum values are provided. The different scenarios are as follows: (a) Low initial population size, mean survival. (b) High initial population size, mean survival. (c) Low initial population size, high survival. (d) High initial population size, high survival. (e) Low initial population size, low survival. (f) High initial population size, low survival

**Figure 4:**
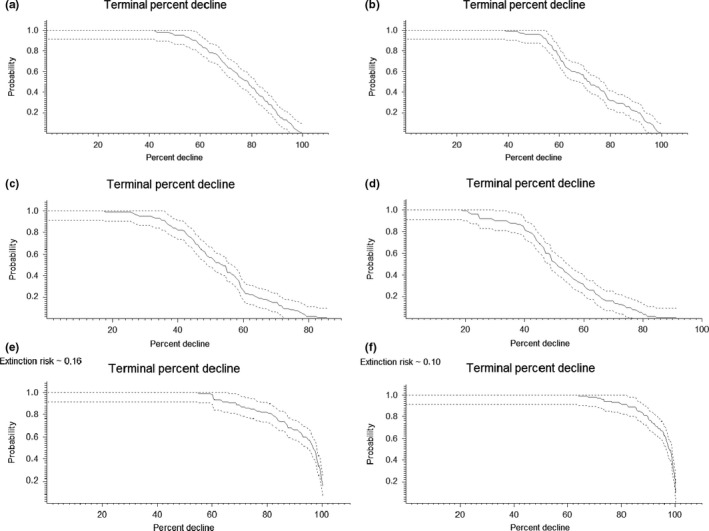
The terminal percent decline is the probability that the metapopulation abundance will have declined by a specific percentage at the end of the simulations. It thus depicts the risk that the final abundance will be less numerous than the original population, and by how much it is likely to drop. For example, in (a), there is an 80% risk of a 60% population decline and a 20% risk of a 90% decline. The extinction risk is noted above the graph when estimated to be >0. The different scenarios are as follows: (a) Low initial population size, mean survival. (b) High initial population size, mean survival. (c) Low initial population size, high survival. (d) High initial population size, high survival. (e) Low initial population size, low survival. (f) High initial population size, low survival

**Figure 5:**
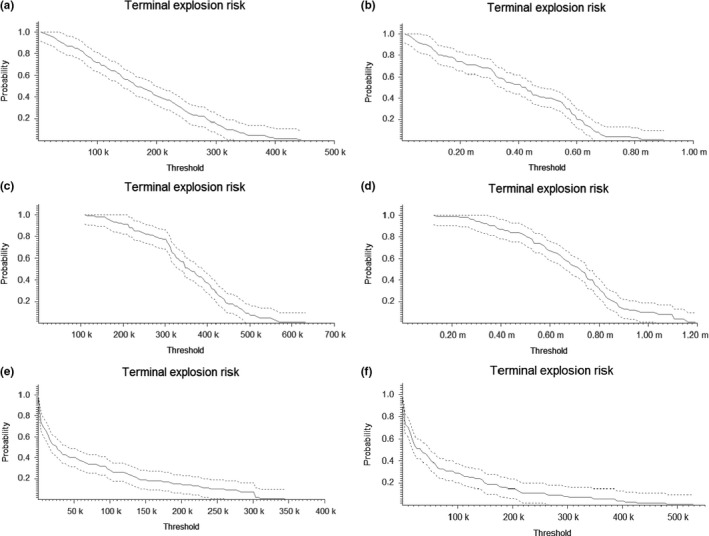
The terminal explosion risk describes the probability that the metapopulation abundance will end up above a specific threshold at the end of the simulations. The different scenarios are as follows: (a) Low initial population size, mean survival. (b) High initial population size, mean survival. (c) Low initial population size, high survival. (d) High initial population size, high survival. (e) Low initial population size, low survival. (f) High initial population size, low survival

The number of populations remaining extant for more time steps increased as the survival rates increased (Figure 3). Populations further to the south had a greater chance of remaining extant than populations further to the north (with the exception of populations 6–11 which were extant more often than extinct). The terminal percent decline is the risk that population abundance will drop by a certain percentage after 100 years, while the terminal explosion risk is the probability that population abundance will surpass a certain threshold after 100 years. Looking at the terminal percent decline, we found a great risk of population decline (Figure 4). At high survival rate, the metapopulation abundance is likely to drop by 20% and has a 50% risk of declining by 55% (Figure 4c,d). At average survival rates, the population will likely drop by 40% and has a 50% risk of declining by 75% (Figure 4a,b). At low survival rates, the European population of Little Owls has a 10% risk of overall extinction and is likely to experience a 60% decline, with a 50% risk of a decline of 90% or more in overall abundance (Figure 4e,f). The terminal explosion risk showed that both the initial population size and the survival rate affect the threshold value (Figure 5).

## 4 DISCUSSION

The already declining population of Little Owls in Europe is likely to continue declining over the next 100 years. The Little Owl prefers warmer areas where the average annual rainfall is low and avoids areas with high tree cover, sparsely vegetated areas, and grassland. Populations further to the north, where the climate is colder, are more likely to decline and disappear than populations further to the south, and the Turkish population will remain the population stronghold over the next 100 years. In order to be genetically viable and minimize the loss of genetic diversity, the individual populations should have a carrying capacity of at least 1,000–30,000 individuals, depending on the actual mortality rates of the populations.

### 4.1 Genetic viability

The PVA in VORTEX suggested that not all populations in Europe are likely to remain extant and retain 99% of the initial genetic variability over the next 100 years. The Balkan population was genetically viable, as were the Spanish and the Italian populations. The Portuguese and Danish populations were not viable in the long run.

The northern European population is estimated to count a maximum of 118,000 individuals. The population is therefore vulnerable to losing a significant amount of genetic diversity. A part of the northern population has already been investigated for loss of genetic diversity, namely the Danish population. A low historic genetic variability was found, and an even lower genetic variability was found in the population at present (Pertoldi et al., 2012). Further, PVA on the Danish population predicts it to perish within the near future (Andersen et al., 2015). An increased mortality level decreased the stochastic growth rate by approximately a factor 10. It would thus be very useful to determine the actual mortality rate of the Portuguese population, in order to determine the actual state of the population. The Portuguese population is at present considered to be stable but fluctuating in size (BirdLife International, 2015).

As the Balkan population counts 24,000–65,000 individuals, the population can be considered genetically viable. The Balkan population consists of the Cypriote population of 4,000–10,000 individuals, the Greek population of 5,000–15,000 individuals, and the Romanian population of 15,000–40,000 individuals (BirdLife International, 2015). Even if considering the low population estimates, both the Romanian and the Greek populations are likely to maintain their genetic diversity if managed as demographically independent populations. The Cypriote population, however, might not in itself be considered a genetically viable management unit if counting only 4,000 individuals. Being an island population located approximately 75 km from mainland Europe, the Cypriote population is effectively isolated from the mainland population. Nevertheless, as the population has survived on the island for centuries, long‐term genetic drift is not likely to pose a great threat to the population. The Italian populations can be considered genetically viable independently of each other and be managed as separate demographic units without risking the loss of a significant amount of genetic diversity. The Spanish population can be considered both genetically viable and demographically independent concerning population management. The MVP(K)s presented here are in line with the MVP(K) found for 28 species of birds, averaging 6,667 individuals (Reed, O'Grady, Brook, Ballou, & Frankham, 2003). Reed et al. (2003), however, did not consider genetic viability but only extinction risk and defined MVP as the minimum K that would decrease the risk of extinction below 1%. A study on Harbor Seals *Phoca vitulina* found the MVP(K) required to maintain 95% genetic diversity to be 75 individuals, while 200 individuals are required to maintain 99% genetic diversity (Olsen et al., 2014). They found that 1,000 individuals are required to ensure a 1% or less risk of extinction.

### 4.2 Habitat suitability

Both climatic factors and landscape structure affected the large‐scale distribution of the Little Owl. This result conforms well to population studies that have found that the reproductive output of Little Owls is limited by excessive rainfall, which may relate to decreased availability of large insects in the breeding season (Bultot et al., 2001; Nieuwenhuyse et al., 2008; Tome et al., 2008). Buildup urban areas have also previously been found to correlate positively with the presence of Little Owl, although only when the buildup areas were at a low intensity (Nieuwenhuyse & Bekaert, 2002).

The Little Owl is associated with the agricultural landscape. Surprisingly, either arable land, mixed agriculture, or permanent crops had a significant influence on the spatial distribution of the Little Owl on a European scale. Previous studies found that both orchards and meadows had a positive influence on the presence of Little Owl (Nieuwenhuyse & Bekaert, 2002; Nieuwenhuyse et al., 2008; Zabala et al., 2006). Increasing tree cover did on the other hand affect the Little Owl negatively, which also conforms well with several investigations showing that Little Owls avoid forested areas (Gottschalk et al., 2011; Nieuwenhuyse et al., 2008; Sunde, Thorup, Jacobsen, & Rahbek, 2014), possibly because of increased predation risk from other raptorial birds such as Tawny Owls (*Strix aluco*; Michel, Jiménez‐Franco, Naef‐Daenzer, & Grüebler, 2016).

The predictive power of the GLM was relatively low, which resulted in a habitat suitability model that did not register the nuance in the highly suitable areas. This might be related to the large scale and large range at which the regression model was built. It could also be due to the scale at which the Little Owl is registered is larger than Little Owl home range. A study on Eurasian Eagle Owls, *Bubo bubo,* examined the distribution of the owl on three spatial scales (Martínez, Serrano, & Zuberogoitia, 2003). A GLM was built for each scale, and Martínez et al. (2003) found that their models were best at predicting the presence/absence of the Eurasian Eagle Owl at home range scale and below. The model build at landscape scale only accounted for 25.97% of the deviance in the distribution. The Eurasian Eagle Owl depended more on small‐scale features such as nesting and feeding grounds than on landscape ecology (Martínez et al., 2003). This could be the case for the Little Owl as well. As an obligate cavity breeder, the Little Owl can only settle in areas where nesting cavities are present. At a large scale, it was not possible to include the presence of nesting cavities in the analysis. A study of habitat preference of the Little Owl and the Long‐eared Owl *Asio otus* in Spain also looked at different scales (Martínez & Zuberogoitia, 2004). Again, the presence of both species was best described at the nest‐site or home range scale, whereas the landscape scale model had the least predictive power. The presence of Little Owl was especially correlated with arid plantations, which is where the Little Owl primarily finds nesting cavities in this area (Martínez & Zuberogoitia, 2004), further confirming that Little Owl distribution might be closely linked to features on a home range scale rather than landscape scale. Throughout its range, the Little Owl is found within different habitat types (Nieuwenhuyse et al., 2008). The incidence that the Little Owl prefers different habitat types with different areas of the study range might also explain the low explanatory power of the model, and it would be interesting to see if the analysis performed better on a smaller part of the range.

Only little over 20% of the variation in Little Owl distribution could be explained by the GLM on a macroscale using landscape and climatic factors. This might be because habitat preference of owl species is best described at a home range scale within a smaller geographical range (Martínez & Zuberogoitia, 2004; Martínez et al., 2003). Thus, when wanting to describe habitat preference and to further manage and conserve owls, it is important to look at a smaller scale and range. The macroscale approach did not catch important parts of the landscape features important to the Little Owl; therefore, it is advisable to work at a microscopic landscape scale.

### 4.3 Population viability and spatial distribution

Most farmland birds are highly mobile in contrast to the modestly mobile Little Owl. Here, population viability was modeled on a continental scale for a modestly mobile vertebrate species vulnerable to isolation and fragmentation. The populations toward the south were more likely to stay occupied and had an overall higher final population abundance than populations further to the north. No matter the initial population size and survival rate the population will decline by at least 20% in the best case scenario and 60% in the worst case scenario. At high survival rates, roughly one‐fourth of the subpopulations had gone extinct by the end of the 100 years, while a low survival resulted in close to half of the subpopulation being extinct. Altering the mortality also affected the local occupancy, with more patches being occupied for longer at high survival rates. Present population declines and disappearances have been explained by decreasing habitat quality and lack of food (Nieuwenhuyse et al., 2008; Thorup et al., 2010; Zmihorski et al., 2006, 2009). Being associated with farmland habitats, the declining population trend is not surprising. Whereas many species of birds linked to a forest habitat have increased over the last decades, birds associated with farmland areas have largely declined (Fuller, 2000; Klvanova, Vorisek, Gregory, Van Strien, & Meyling, 2009, PECBMS, 2007; Reif, Storch, Voříšek, Šťastný, & Bejček, 2008). Within Europe, the greatest decline in farmland species between 1980 and 2005 was found in northern Europe, followed by western Europe, while the least decline was seen in southern Europe (PECBMS, 2007). The decline is more severe in western European countries compared to eastern European countries formerly part of the USSR (Donald, Green, & Heath, 2001). Several widespread farmland species generally considered common (large range and listed as Safe by the IUCN) have experienced dramatic declines, including the Willow Tit *Parus montanus* and the Lesser Spotted Woodpecker *Dendrocopos minor* (PECBMS, 2007). And as the agricultural production is expected to double from year 2000 to 2050, the intensification of agricultural lands is likely to continue (Tilman, 1999). It is therefore of utmost importance to identify indicator species whose population trend reflect the trend of the overall farmland biodiversity. With its large dietary range (including everything in the size range from ants to young rabbits; Nieuwenhuyse et al., 2008) and its dependency on a multitude of alternative foraging habitats over the years and as weather change (Sunde et al., 2014), the Little Owl is likely a good indicator species of abundance and diversity of these prey in farmland habitats.

### 4.4 Implications for management and conservation status

The overall abundance of Little Owls in Europe is predicted to decline over the next 100 years. Both VORTEX and RAMAS/GIS found that populations further to the north are more likely to decline and potentially go extinct than populations further to the south. This is in agreement with the results from the habitat suitability analysis that annual mean temperature is a positive predictor of Little Owl presence. When comparing with population data from the BirdLife International (2015), this is in agreement with the current estimated long‐term population trends. Here, southeastern populations were estimated to be overall stable, while most northwestern populations were expected to decline. The data presented here suggest that the declining population trend will continue and affect populations throughout the entire distributional range. It is, however, important to stress that the results presented here only are as valid as the input parameters. They should therefore only be used as guidelines with regards to general trends.

In these simulations, the European population of Little Owls was considered a complex of interconnected populations and subpopulations of which some might persist as true metapopulations. This might be true for parts of the European population (Nieuwenhuyse et al., 2008). But in central and western Europe, the populations are likely subdivided into smaller and potentially isolated units than the ones used in the models. Therefore, the results might be overly optimistic with regards to population size and overstate the role of dispersal and genetic exchange between de facto isolated populations.

When managing populations of Little Owl within Europe, population size is an important factor if wanting to preserve genetic diversity and evolutionary potential. Depending on the actual survival rates of the population, our analysis indicated that there must be capacity for a minimum of 1,000 individuals to preserve 95% genetic diversity over 100 years, and between 4,500–30,000 individuals if 99% genetic diversity is to be maintained. Several populations within Europe count <1,000 individuals (including the Danish, Polish, Austrian, and Swiss populations), and few are estimated to count more than 30,000 (BirdLife International, 2015). In order to prevent genetic loss from the smallest and most isolated populations, concrete actions to increase gene flows (of which active translocation of individuals between populations would be the most simple but also the most artificial and increase in population size or connection of populations through habitat improvements the most costly but least artificial method) might therefore be necessary.

## CONFLICT OF INTEREST

None declared.

## AUTHORS’ CONTRIBUTION

LHA, VL, and CP planned the project and set the aims. IP and CP provided the genomic data used in the analyses. IP performed molecular analyses. LHA conducted the analyses in ArcGIS, RAMAS/GIS, and VORTEX, analyzed the data, and made the first draft to the manuscript, under the supervision of VL and CP. PS provided expert knowledge on the Little Owl and contributed in improving the data analysis and overall manuscript quality. All authors revised and commented on the manuscript and its contents.
