# Supplementary figures and images for: Using population viability analysis, genomics, and habitat suitability to forecast future population patterns of Little Owl Athene noctua across Europe
Source: Ecol Evol. 2017 Nov 12;7(24):10987–1001. doi: 10.1002/ece3.3629 (PMC5743613; doi:10.1002/ece3.3629)

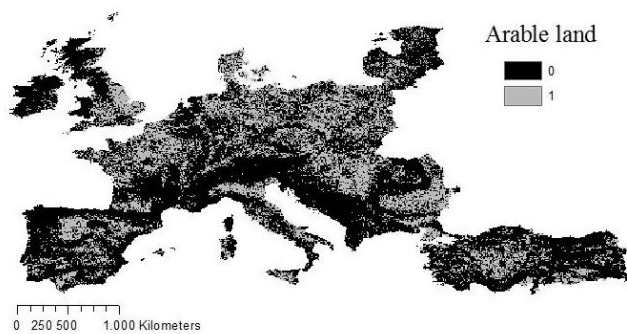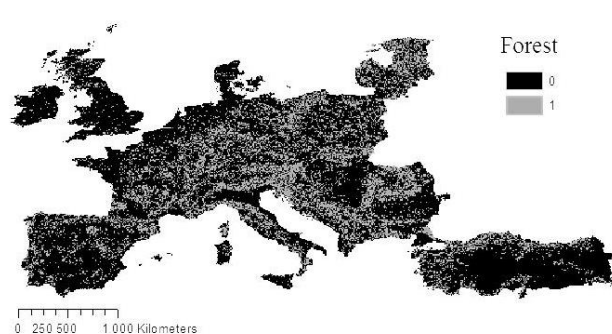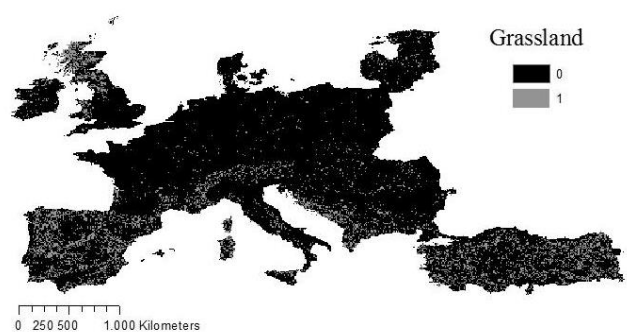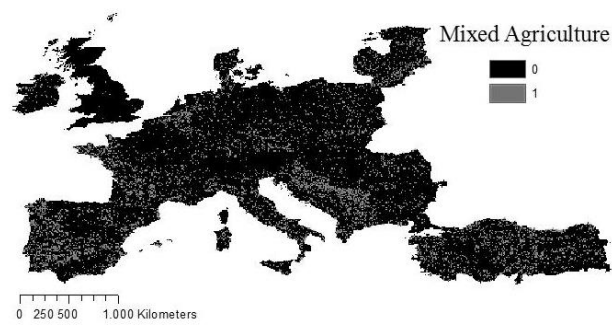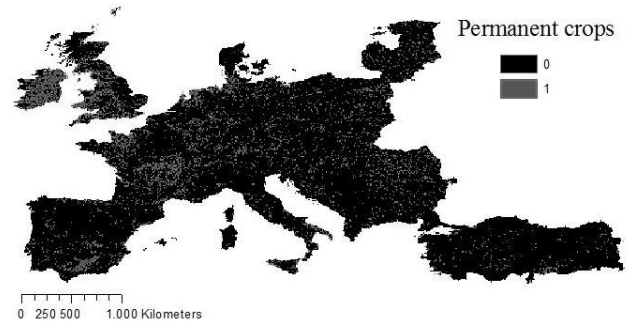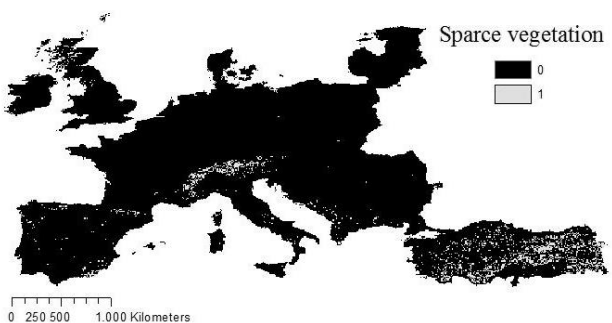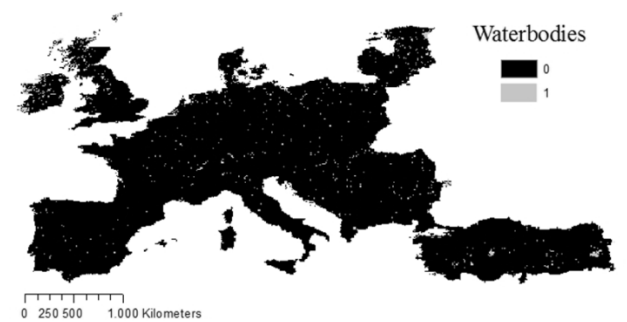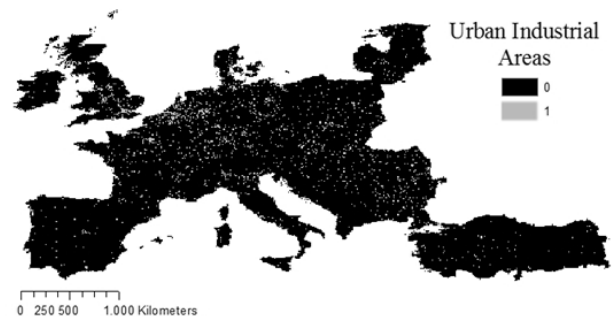

Supplement: Supplementary file 1 [file ECE3-7-10987-s001.pdf]

## Dispersal-distance

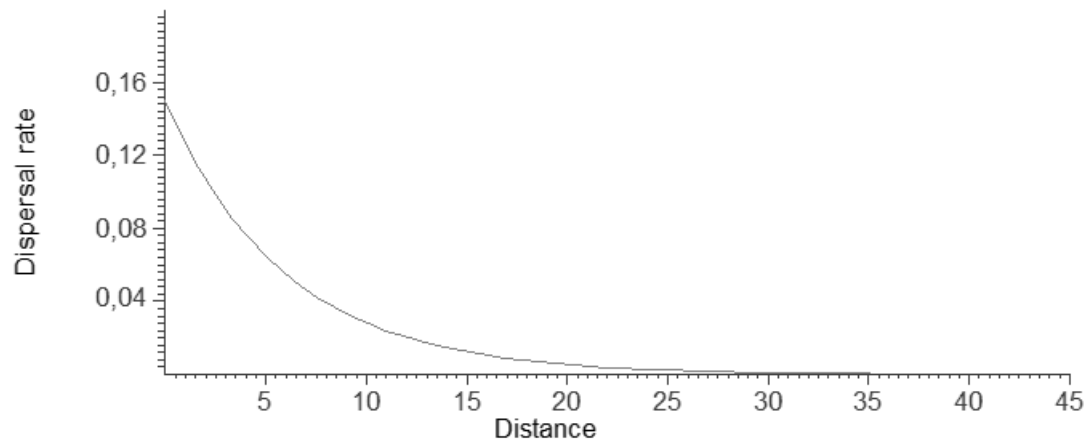

Supplement: Supplementary file 2 [file ECE3-7-10987-s002.pdf]

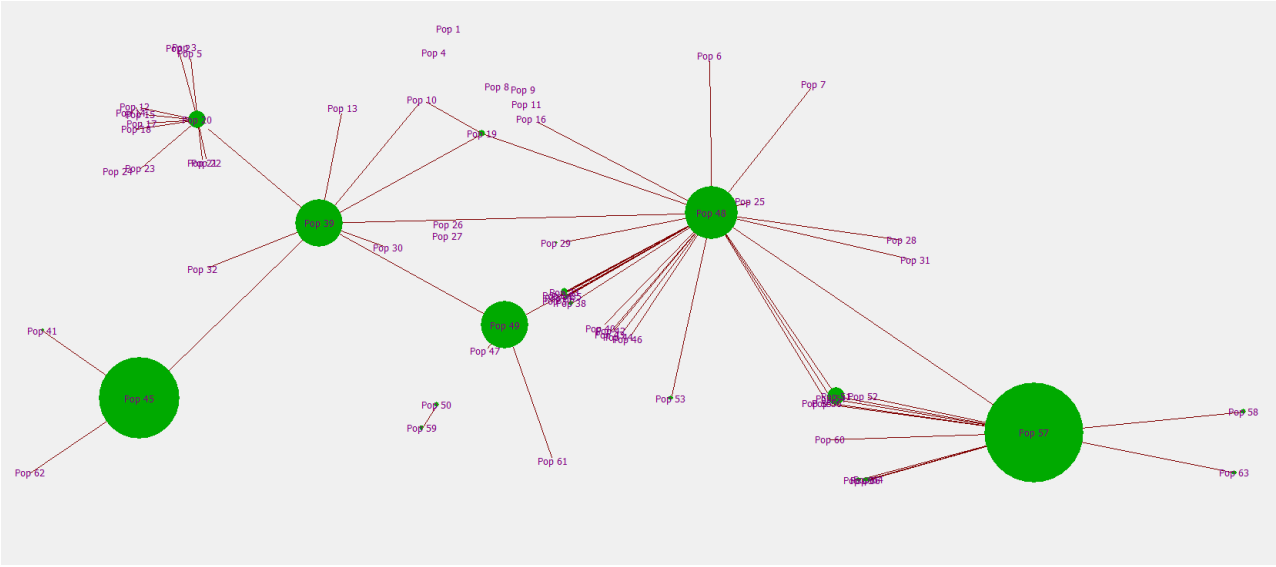

Supplement: Supplementary file 3 [file ECE3-7-10987-s003.pdf]

A)

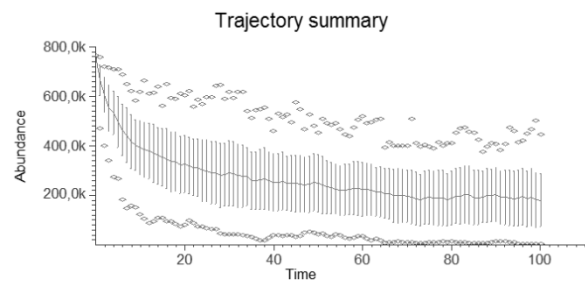

B)

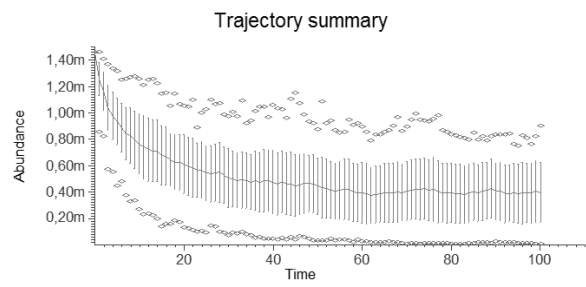

C)

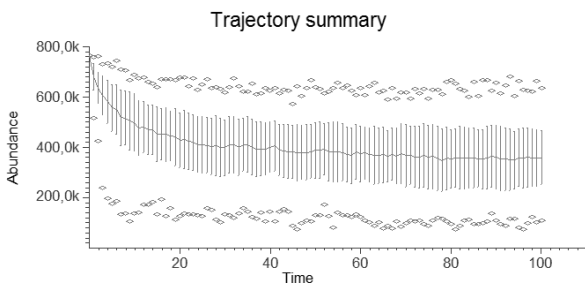

D)

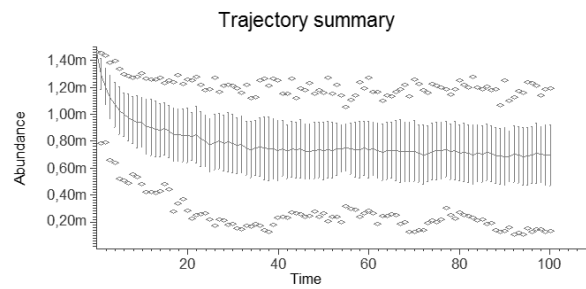

E)

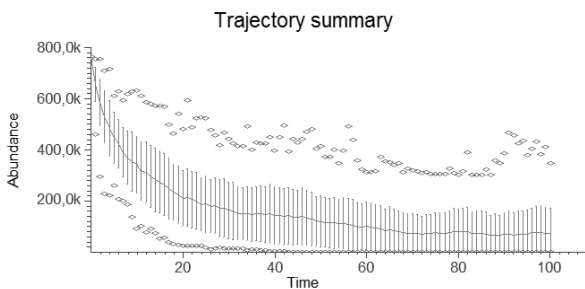

F)

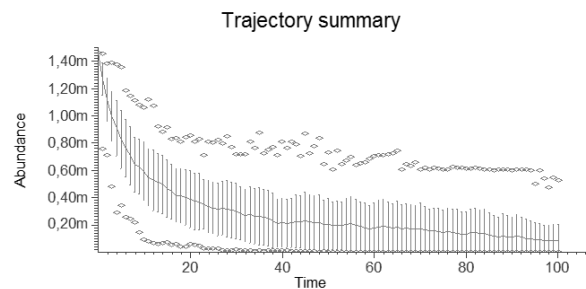

Supplement: Supplementary file 4 [file ECE3-7-10987-s004.pdf]
