# Supplementary material for: Using population viability analysis, genomics, and habitat suitability to forecast future population patterns of Little Owl Athene noctua across Europe
Source: Ecol Evol. 2017 Nov 12;7(24):10987–1001. doi: 10.1002/ece3.3629 (PMC5743613; doi:10.1002/ece3.3629)
Supplement: Supplementary file 5 [file ECE3-7-10987-s005.docx]

| Table S1. The carrying capacity for each of the regions included in the VORTEX analysis. The data on the area of each region was retrieved from The World Bank. Data on density was retrieved from (Nieuwenhuyse et al., 2008, Sunde et al., 2009). When more than one density was listed for a region, the mean density was calculated. K was calculated as area times density. The carrying capacity was rounded to the nearest 50. As no densities exist for Greece, Romania or Cyprus, an estimated K of 250,000 individuals is used. *Due to limitations of the program a K of 100,000 was simulated. | | | |  |
| --- | --- | --- | --- | --- |
| Region | Area (km^2^) | Density (breeding pairs/km^2^) | K (individuals) | |
| France (FR) | 547,557 | 0.54 | 586,800 | |
| Denmark (DK) | 42,430 | 0.05 | 4,200 | |
| The Netherlands (NL) | 33,720 | 1.0 | 67,450 | |
| FR-DK-NL | 623,707 | - | 658,450 | |
| Portugal (PT) | 91,590 | 4.66 | 852,700 | |
| Spain (ES) | 499,880 | 1.35 | 1,353,300* | |
| Italy (IT) | 294,140 | 0.75 | 438,500* | |
| Greece (GR) | 128,900 |  |  | |
| Romania (RM) | 230,170 |  |  | |
| Cyprus (CP) | 9,240 |  |  | |
| GR-RM-CP | 368,310 | - | 100,000 | |
