# Supplementary material for: Using population viability analysis, genomics, and habitat suitability to forecast future population patterns of Little Owl Athene noctua across Europe
Source: Ecol Evol. 2017 Nov 12;7(24):10987–1001. doi: 10.1002/ece3.3629 (PMC5743613; doi:10.1002/ece3.3629)
Supplement: Supplementary file 6 [file ECE3-7-10987-s006.docx]

| Table S2. An overview of survival rates as reported in the literature. | | |
| --- | --- | --- |
| Population | Juvenile survival rate | Adult survival rate |
| Denmark | 15% (Thorup et al., 2010) | 61% (Thorup et al., 2010) |
| Netherlands | 25.8% (Gouar et al., 2010);  29.9% (Exo, 1992) | 75.3% (Gouar et al., 2010);  70.8% (Exo, 1992);  62.1% (Exo, 1992) |
| Germany | 51.1% (Nieuwenhuyse et al., 2008); 13% (Schaub et al., 2006) | - |
| Switzerland | 13% (Schaub et al., 2006);  26% (Exo, 1992) | 65.8% (Schaub et al., 2006) |
