# Supplementary material for: Using population viability analysis, genomics, and habitat suitability to forecast future population patterns of Little Owl Athene noctua across Europe
Source: Ecol Evol. 2017 Nov 12;7(24):10987–1001. doi: 10.1002/ece3.3629 (PMC5743613; doi:10.1002/ece3.3629)
Supplement: Supplementary file 7 [file ECE3-7-10987-s007.docx]

| Table S3. A list of what the different data-categories from the CORINE dataset include (EuropeanEnvironmentAgency, 2013). | | |
| --- | --- | --- |
| Name | Land cover code | Description |
| URB (Urban and industrial areas) | 111-124 | Continuous urban fabric, Airports, Port areas, Road and rail networks and associated land, Discontinuous urban fabric, Industrial or commercial units |
| ARA (Arable land | 211-213 | Non-irrigated arable land, Permanently irrigated land, Rice fields |
| PCR (Permanent crops) | 221-231 | Vineyards, Fruit trees and berry plantations, Pastures, Olive groves |
| MAG (Mixed agriculture) | 241-244 | Annual crops associated with permanent crops, Agro-forestry areas, Land principally occupied by agriculture, with significant areas of natural vegetation, Complex cultivation patterns |
| FOR (Forest) | 311-313 | Broad-leaved forest, Coniferous forest, Mixed forest |
| GRA (Grassland) | 321-324 | Natural grasslands, Moors and heathland, Transitional woodland-shrub, Sclerophyllous vegetation |
| SVE (Sparse or no vegetation) | 331-335 | Beaches, dunes, sands, Bare rocks, Sparsely vegetated areas, Burnt areas, Glaciers and perpetual snow |
| WAT (Water bodies) | 411-412, 421-423, 511-512, 521-523 | Inland marshes, Intertidal flats, Peat bogs, Water courses, Water bodies, Salt marshes, Coastal lagoons, Salines, Sea and ocean, Estuaries |
