# Supplementary material for: Using population viability analysis, genomics, and habitat suitability to forecast future population patterns of Little Owl Athene noctua across Europe
Source: Ecol Evol. 2017 Nov 12;7(24):10987–1001. doi: 10.1002/ece3.3629 (PMC5743613; doi:10.1002/ece3.3629)
Supplement: Supplementary file 8 [file ECE3-7-10987-s008.docx]

| Table S4. The subpopulations of the metapopulation simulated in RAMAS Metapop, and the initial population sizes (N). | | | | |
| --- | --- | --- | --- | --- |
| Population | Approximate location | Low N | High N |  |
| 1 | Denmark | 50 | 50 |  |
| 2 | Great Britain | 100 | 100 |  |
| 3 | Great Britain | 100 | 100 |  |
| 4 | Denmark | 20 | 20 |  |
| 5 | Great Britain | 100 | 100 |  |
| 6 | Lithuania | 100 | 100 |  |
| 7 | Lithuania | 10 | 10 |  |
| 8 | Germany | 200 | 200 |  |
| 9 | Germany | 200 | 200 |  |
| 10 | Western Europe (Netherlands, Germany, France, Belgium) | 200 | 200 |  |
| 11 | Germany | 200 | 200 |  |
| 12 | Great Britain | 100 | 100 |  |
| 13 | Western Europe (Netherlands, Germany, France, Belgium) | 100 | 100 |  |
| 14 | Great Britain | 100 | 100 |  |
| 15 | Great Britain | 100 | 100 |  |
| 16 | Germany | 200 | 200 |  |
| 17 | Great Britain | 100 | 100 |  |
| 18 | Great Britain | 100 | 100 |  |
| 19 | Germany | 2,000 | 2,500 |  |
| 20 | Great Britain | 10,000 | 11,000 |  |
| 21 | Great Britain | 100 | 100 |  |
| 22 | Great Britain | 100 | 100 |  |
| 23 | Great Britain | 100 | 100 |  |
| 24 | Great Britain | 100 | 100 |  |
| 25 | Eastern Europe (Poland, Albania, Kosovo, Latvia, Lithuania, FYROM, Moldova, Montenegro, Romania, Serbia, Slovakia, Slovenia) | 100 | 100 |  |
| 26 | Austria | 140 | 170 |  |
| 27 | Austria | 140 | 170 |  |
| 28 | Eastern Europe (Poland, Albania, Kosovo, Latvia, Lithuania, FYROM, Moldova, Montenegro, Romania, Serbia, Slovakia, Slovenia) | 100 | 100 |  |
| 29 | Czech Republic | 500 | 1000 |  |
| 30 | Switzerland | 160 | 220 |  |
| 31 | Eastern Europe (Poland, Albania, Kosovo, Latvia, Lithuania, FYROM, Moldova, Montenegro, Romania, Serbia, Slovakia, Slovenia) | 100 | 100 |  |
| 32 | Western Europe (Netherlands, Germany, France, Belgium) | 100 | 100 |  |
| 33 | Croatia | 2,000 | 3000 |  |
| 34 | Eastern Europe (Poland, Albania, Kosovo, Latvia, Lithuania, FYROM, Moldova, Montenegro, Romania, Serbia, Slovakia, Slovenia) | 100 | 100 |  |
| 35 | Croatia | 1,000 | 1,500 |  |
| 36 | Eastern Europe (Poland, Albania, Kosovo, Latvia, Lithuania, FYROM, Moldova, Montenegro, Romania, Serbia, Slovakia, Slovenia) | 100 | 100 |  |
| 37 | Eastern Europe (Poland, Albania, Kosovo, Latvia, Lithuania, FYROM, Moldova, Montenegro, Romania, Serbia, Slovakia, Slovenia) | 100 | 100 |  |
| 38 | Croatia | 1,000 | 1500 |  |
| 39 | Western Europe (Netherlands, Germany, France, Belgium, | 70,000 | 152,880 |  |
| 40 | Eastern Europe (Poland, Albania, Kosovo, Latvia, Lithuania, FYROM, Moldova, Montenegro, Romania, Serbia, Slovakia, Slovenia) | 100 | 100 |  |
| 41 | Southwestern Europe (Portugal, Spain) | 500 | 500 |  |
| 42 | Eastern Europe (Poland, Albania, Kosovo, Latvia, Lithuania, FYROM, Moldova, Montenegro, Romania, Serbia, Slovakia, Slovenia) | 100 | 100 |  |
| 43 | Eastern Europe (Poland, Albania, Kosovo, Latvia, Lithuania, FYROM, Moldova, Montenegro, Romania, Serbia, Slovakia, Slovenia) | 100 | 100 |  |
| 44 | Eastern Europe (Poland, Albania, Kosovo, Latvia, Lithuania, FYROM, Moldova, Montenegro, Romania, Serbia, Slovakia, Slovenia) | 100 | 100 |  |
| 45 | Southwestern Europe (Portugal, Spain) | 180,000 | 354,000 |  |
| 46 | Eastern Europe (Poland, Albania, Kosovo, Latvia, Lithuania, FYROM, Moldova, Montenegro, Romania, Serbia, Slovakia, Slovenia) | 0 | 0 |  |
| 47 | Italy | 0 | 0 |  |
| 48 | Eastern Europe (Poland, Albania, Kosovo, Latvia, Lithuania, FYROM, Moldova, Montenegro, Romania, Serbia, Slovakia, Slovenia) | 85,000 | 175,120 |  |
| 49 | Italy | 70,000 | 120,000 |  |
| 50 | Italy | 1,000 | 1,000 |  |
| 51 | Turkey | 10,000 | 20,000 |  |
| 52 | Turkey | 100 | 100 |  |
| 53 | Eastern Europe (Poland, Albania, Kosovo, Latvia, Lithuania, FYROM, Moldova, Montenegro, Romania, Serbia, Slovakia, Slovenia) | 1,000 | 2,000 |  |
| 54 | Turkey | 100 | 100 |  |
| 55 | Turkey | 100 | 100 |  |
| 56 | Turkey | 0 | 0 |  |
| 57 | Turkey | 300,000 | 600,000 |  |
| 58 | Turkey | 1,000 | 1,000 |  |
| 59 | Italy | 1,000 | 1,000 |  |
| 60 | Turkey | 100 | 100 |  |
| 61 | Italy | 100 | 100 |  |
| 62 | Southwestern Europe (Portugal, Spain) | 200 | 200 |  |
| 63 | Turkey | 1,000 | 1,000 |  |
| 64 | Turkey | 1,000 | 1,000 |  |
| 65 | Turkey | 1,000 | 1,000 |  |
| 66 | Turkey | 1,000 | 1,000 |  |
|  | SUM (individuals) | 744,720 | 1,456840 |  |
