# Supplementary material for: Using population viability analysis, genomics, and habitat suitability to forecast future population patterns of Little Owl Athene noctua across Europe
Source: Ecol Evol. 2017 Nov 12;7(24):10987–1001. doi: 10.1002/ece3.3629 (PMC5743613; doi:10.1002/ece3.3629)
Supplement: Supplementary file 9 [file ECE3-7-10987-s009.docx]

| Table S5. The observed clutch size and the expected clutch size calculated on basis of the formula in Exo (1992). When possible, the observed mean clutch size was used in the VORTEX simulations. If the mean clutch size of a population has not been reported, the average calculated clutch size was used in the VORTEX simulations. The mean clutch size was used in the RAMAS/GIS simulations. Only recorded clutch sizes were used in calculating the mean. The largest number of eggs recorded in a nest is 10 (Nieuwenhuyse et al., 2008). ^1^The mean no of fledglings was 0.6-2.3 depending on habitat type and year. ^2^The mean clutch size was estimated by the numbers in fig. 2B. ^3^Calculated from the numbers in Fig. 4. The mean number of fledglings was 2.39±SD1.15. | | | | | | | |  |
| --- | --- | --- | --- | --- | --- | --- | --- | --- |
| Country | Latitude (LT) | LT clutch size | Longitude (LG) | LG clutch size | Mean | SD | Observed mean clutch size (±SD if recorded) | |
| Portugal | 38.46 | 5.47 | -9.90 | 2.72 | 4.10 | 1.95 | 3.3±1.2^1^ (Tome et al., 2008) | |
| Spain | 40.00 | 5.29 | -4.00 | 3.13 | 4.21 | 1.53 | ~4.4^2^ (Parejo et al., 2012) | |
| France | 46.00 | 4.57 | 2.00 | 3.55 | 4.06 | 0.72 | 3.67 (Nieuwenhuyse et al., 2008) | |
| Denmark | 56.00 | 3.37 | 10.00 | 4.11 | 3.74 | 0.52 | 3.84^3^ (Jacobsen, 2006) | |
| Netherlands | 52.23 | 3.82 | 4.55 | 3.72 | 3.78 | 0.07 | 3.82 (Nieuwenhuyse et al., 2008) | |
| Romania | 46.00 | 4.57 | 25.00 | 5.16 | 4.87 | 0.42 | 5.24 (Nieuwenhuyse et al., 2008) | |
| Greece | 34.00 | 6.01 | 22.00 | 4.95 | 5.48 | 0.75 |  | |
| Cyprus | 35.00 | 5.89 | 33.00 | 5.72 | 5.81 | 0.12 |  | |
| Italy | 42.50 | 4.99 | 12.50 | 4.29 | 4.64 | 0.50 |  | |
| Russia |  |  |  |  |  |  | 4.87 (Nieuwenhuyse et al., 2008) | |
| Belgium | 50.50 | 4.03 | 4.47 | 3.72 |  |  | 3.36; 3.18; 3.85 (Nieuwenhuyse et al., 2008) | |
| Hungary | 47.16 | 4.43 | 19.50 | 4.78 |  |  | 5 (Nieuwenhuyse et al., 2008) | |
| Germany | 51.16 | 3.95 | 10.45 | 4.14 |  |  | 4.15; 3.51; 3.71; 3.65; 3.85; 4.13; 3.60; 4.11; 4.42; 3.61 (Nieuwenhuyse et al., 2008) | |
| Switzerland | 46.81 | 4.78 | 8.23 | 3.99 |  |  | 3.70; 3.12 (Nieuwenhuyse et al., 2008) | |
| Britain | 55.38 | 3.44 | -3.44 | 3.17 |  |  | 2.65; 3.59 (Glue and Scott, 1980, Nieuwenhuyse et al., 2008) | |
| Front-Caucasian area |  |  |  |  |  |  | 4.78±0.22 (Nieuwenhuyse et al., 2008) | |
| MEAN |  |  |  |  |  |  | 3.87 | |
